# Defining the blanks – Pharmacochaperoning of SLC6 transporters and ABC transporters^?^

**DOI:** 10.1016/j.phrs.2013.11.009

**Published:** 2014-05

**Authors:** Peter Chiba, Michael Freissmuth, Thomas Stockner

**Affiliations:** a Institute of Medical Chemistry, Center of Pathobiochemistry and Genetics, Waehringer Strasse 10, A-1090 Vienna, Austria; b Institute of Pharmacology, Center of Physiology and Pharmacology, Medical University of Vienna, Waehringer Strasse 13a, A-1090 Vienna, Austria

**Keywords:** ABC transporters, ATP-binding cassette containing transporters, DAT, dopamine transporter, ER, endoplasmic reticulum, GAT, GABA transporter with 4 isoforms (GAT-1, GAT-2, GAT-3, GAT-4), NBD, nucleotide binding domains, NET, norepinephrine transporter, SLC6, solute carrier-6, SERT, serotonin transporter, ABC transporters, Chaperones, ER export, Folding-deficient mutants, Pharmacochaperoning, SLC6 transporters

## Abstract

SLC6 family members and ABC transporters represent two extremes: SLC6 transporters are confined to the membrane proper and only expose small segments to the hydrophilic milieu. In ABC transporters the hydrophobic core is connected to a large intracellular (eponymous) ATP binding domain that is comprised of two discontiguous repeats. Accordingly, their folding problem is fundamentally different. This can be gauged from mutations that impair the folding of the encoded protein and give rise to clinically relevant disease phenotypes: in SLC6 transporters, these cluster at the protein–lipid interface on the membrane exposed surface. Mutations in ABC-transporters map to the interface between nucleotide binding domains and the coupling helices, which provide the connection to the hydrophobic core. Folding of these mutated ABC-transporters can be corrected with ligands/substrates that bind to the hydrophobic core. This highlights a pivotal role of the coupling helices in the folding trajectory. In contrast, insights into pharmacochaperoning of SLC6 transporters are limited to monoamine transporters – in particular the serotonin transporter (SERT) – because of their rich pharmacology. Only ligands that stabilize the inward facing conformation act as effective pharmacochaperones. This indicates that the folding trajectory of SERT proceeds via the inward facing conformation. Mutations that impair folding of SLC6 family members can be transmitted as dominant or recessive alleles. The dominant phenotype of the mutation can be rationalized, because SLC6 transporters are exported in oligomeric form from the endoplasmic reticulum (ER). Recessive transmission requires shielding of the unaffected gene product from the mutated transporter in the ER. This can be accounted for by a chaperone-COPII (coatomer protein II) exchange model, where proteinaceous ER-resident chaperones engage various intermediates prior to formation of the oligomeric state and subsequent export from the ER. It is likely that the action of pharmacochaperones is contingent on and modulated by these chaperones.

## Graphical abstract

**Figure:**
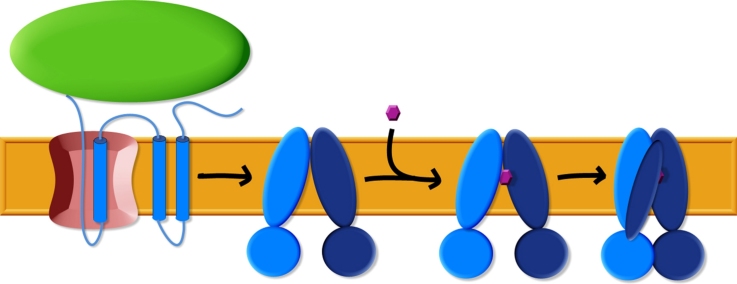

## 1 Introduction

Many (monogenic) diseases result from point mutations that lead to aberrant folding of a protein. In fact, the term molecular medicine can be traced back to the efforts of Max Perutz to solve the structure of hemoglobin and of Linus Pauling and coworkers to understand the defect underlying sickle cell anemia [1]. Folding diseases may be rare by comparison to other widespread afflictions that figure prominently in health care budgets such as diabetes mellitus, obesity and coronary heart disease. However, as pointed out by Sir Archibald Garrod some 75 years ago, major scientific advances have come from the study of rare diseases [2]. Understanding protein folding remains a fascinating challenge, which is succinctly illustrated by the *Gedanken experiment* of Cyrus Levinthal [3]. Its insights can be recapitulated in a back-of-the-envelope calculation: (i) for the sake of simplicity, assume that the two bonds per amino acid, which define the peptide backbone of a protein, can exist in two stable states (e.g., the angles that define an a-helix and ß-strand). (ii) New conformations can be visited in 0.1 ps, i.e. at a rate at which single bonds orient (10^-13^ s^-1^). Hence, a protein can explore 10^13^ states in one second. (iii) Under these assumptions, an average protein of some 400 amino acids has to sample some 2^400^ (˜10^120^) conformational states in the absence of any additional constraint. In other words, exploring these states requires 10^107^ s. It is evident that this is an implausible time frame, for the currently estimated age of the known universe is 4.41 × 10^17^ s (1.4 × 10^10^ years). This discrepancy – referred to as the Levinthal paradoxon – proves that the folded structure of a protein is reached via a limited set of trajectories rather than a random, unbiased search of all possible conformations.

The folding trajectories of polytopic membrane proteins are subject to a number of constraints: (i) insertion of the nascent polypeptide chain into the SEC61 translocon channel restricts the movement of amino acid side chains and limits the search space. (ii) Transmembrane segments exit via a lateral gate of the translocon into lipid bilayer. This gate allows for passage of one or at most two transmembrane segments [4]. (iii) The milieu of the lipid bilayer is anisotropic. The presence of charged residues within the transmembrane segment, for instance, imposes a high energetic cost [5].

Transmembrane helices are typically 21–22 residues long [6]. The conformationally important backbone of a single transmembrane helix has 315–330 degrees of freedom and can – according to the concept outlined above – sample ~10^6^ conformations. Folding of single transmembrane helices in the SEC61 translocon channel occurs on the time scale of peptide chain synthesis or faster. Once the transmembrane helix is inserted into the membrane, the biophysical properties of the membrane environment dramatically restrict this available search space: to a first approximation, (i) the number of accessible conformations is limited to only the all-helical state. (ii) The relevant degrees of freedom are reduced to 4, as the helix can only diffuse in the plane of the membrane (*x* and *y*), rotate along its main axis, and tilt relative to the plain of the membrane. Diffusion of helices that are connected by loops is a slow process. However, the search space is dramatically reduced by the steep drop in the number of degrees of freedom and in the number of accessible conformations.

Pharmacochaperoning is – by definition – linked to the problem of protein folding. The question arises: why should it be of interest to study pharmacochaperoning of ABC-transporters^3^ and SLC6 transporters? From a medical perspective, these two classes of transporters are highly relevant to pharmacotherapy either as drug targets (in particular the SLC6 family members SERT, NET, DAT, GAT-1) or as efflux pumps (ABC-transporters). In addition, mutations in ABC transporters are the cause of monogenic diseases other than the paradigmatic folding disease cystic fibrosis (e.g., cholestasis, gout). In addition, these two classes illustrate the folding problem of dynamic polytopic membrane proteins. They have 12 or more transmembrane segments. They are thought to undergo major conformational changes and thus domain motions during the transport cycle. This raises the question of which conformations are visited by the folding trajectory. Finally, SLC6 transporters and ABC-transporters are representative two extremes: in SLC6 family members, the hydrophobic core comprises the bulk of the protein, intracellular and extracellular segments are small by comparison. This confines the folding problem to the membrane. In contrast, the folding trajectory of ABC-transporters must accommodate both and the folding of the large intracellular domains must be coupled to that of the transmembrane segment. In fact, mutations associated with folding defects of ABC transporters cluster at the interface between the transmembrane domain and the NBDs (see below). This highlights the Achilles heel of ABC transporters in the folding trajectory. Here we argue that experimental approaches to pharmacochaperoning these transporters may provide insights into the folding process or at the very least allow for defining the problem. We also examine the conjecture that commonly used drugs may also act as pharmacochaperones when administered for therapeutic or recreational purposes [7]. Finally, in the long run, pharmacochaperoning may be used to correct folding diseases.

### 1.1 Folding-deficient versions of SLC6 transporters

The human genome encodes 19 SLC6 transporters. SLC6A10 is thought to be a pseudogene. Hence the numbering extends from SLC6A1 (i.e., GAT-1) to SLC6A20 (i.e., the renal imino acid transporter). Human diseases appear to be rarely associated with mutations in these genes. This can be illustrated with the gene encoding GAT1: the NCBI database lists more than 1000 single nucleotide polymorphisms in the human SLCA1 gene. Seventeen of these result in non-synonymous substitutions and thus give rise to coding variants of GAT-1. It is at present not known, if one of these SNPs are related to a clinically relevant phenotype. However, there are some examples of (monogenic) diseases and clinically relevant phenotypes that can be linked to mutations in SLC6-family genes (Table 1); there are also less well-defined phenotypes that may eventually be found to arise from defective folding; pertinent examples are also listed in Table 1.

It is worth noting that mutations in SLC6 family members can be transmitted in both, an autosomal dominant and a recessive manner. It is trivial to understand why recessive alleles only cause symptoms in homozygous (or compound homozygous) individuals: clinical symptoms only appear in the total absence of a transporter.

A dominant negative phenotype can be envisaged, if the mutated transporter forms a complex with the product of the unaffected allele and precludes its surface expression. This is for instance evident in NET and GLYT2, where the mutated versions NET-A457P [8] and GLYT2-S510R [21] retain the corresponding wild type transporter within the cell (see Table 1). SLC6 family members form oligomers [44]. Hence, dominant negative effects can be readily rationalized. However, this model does not account for recessive transmission, which is observed for instance in DAT mutations associated with infantile dystonia/parkinsonism [15,16].

We mapped the residues that cause folding deficiency onto a common structural scaffold, i.e., a recently published hDAT structure [45]. This shows that the mutations are not randomly distributed (Fig. 2, color coded by transporter). The largest group clusters at the protein–lipid interface on the membrane exposed surface of the transporter. Most of these mutations are conservative in amino acid substitution, retaining the hydrophobic property of the residue. Polarity changes are therefore not expected to add a significant destabilizing energy to the folding process. Interactions with other proteins within the membrane are not expected to be severely affected, because dispersive hydrophobic interactions are rather unspecific and because hydrophobicity driven complexes have large interaction surfaces stabilized by cumulative contributions. What could then be at the origin of their effect? Almost all of these disease causing mutations change the helical properties of the mutated residue. It is therefore conceivable that helical stability is affected, which links these mutations directly to the protein folding and stability.

A second group of mutations clusters at helix crossing motifs or affect helix packing, thereby destabilizing the global structure of the transporter. Some mutations linked to diseases are found in water exposed loops. These are all found at critical position of structural motifs. Their mutation is expected to reduce protein stability. One mutation is located close to the Sec24 interaction motif (residue K605N of SERT]). It may affect recognition of SERT by Sec24C.

A small number of mutations are expected to have a strong effect on the conformational equilibrium. This has for example been shown for the completely conserved arginine in the outer vestibule on transmembrane helix 1. In the inward facing state, this arginine forms a salt bridge across the outer vestibule with an aspartate or glutamate in transmembrane helix 10. Hartnup disease that results in dermatitis and seizures is caused by a mutation of this arginine residue to cysteine in the B0AT1 amino acid transporter [39–41]. Conformation affecting mutations will have a dual effect: they ought to impair protein function. In addition, they are likely to be recognized by the quality control machinery of the ER: the mutant proteins are structurally less stable; hence they are predicted to switch readily between states. This makes the protein more susceptible for ubiquitination and degradation.

The effect of substrate binding to the conformational equilibrium has been directly examined in the bacterial homolog LeuTAa [46] using single molecule FRET. The transporter was shown to have a fast conformational exchange between the inward and the outward facing conformation and binding of substrate was found to stabilize the protein in one conformation.

### 1.2 A macroscopic model for pharmacochaperoning of SLC6 family members

**Fig. 1:**
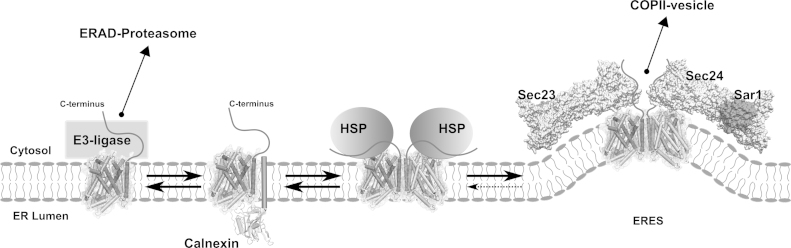
A chaperone-COPII-exchange model for SLC6 family members in the ER. Upon exit from the SEC61 channel (not shown), SLC family members are first shielded by luminal chaperones (shown here as calnexin, CNX) and cytosolic heat-shock proteins (HSP). Release of CNX allows for oligomerization; release of cytosolic heat-shock protein-90 from the C-terminus unmasks the SEC24 binding site in the C-terminus and allos for ER export of oligomeric transporters. If the transporter fails to reach a stable conformation, it is relayed to the ER-associated degradation (ERAD) via the recruitment of an E3-ligase.

**Fig. 2:**
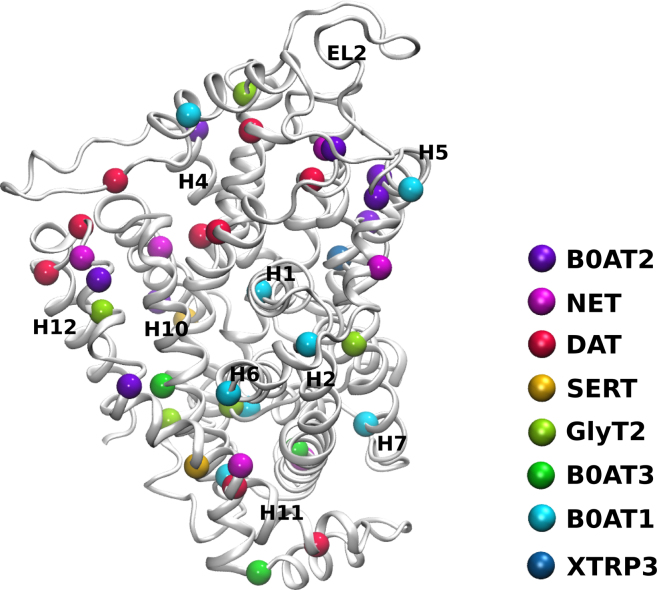
Structural mapping of disease causing mutation that are linked to deficiencies in protein folding. The transmembrane domain DAT is shown as representative member of the SLC6 family from its extracellular site. Residues identified in the SLC6A transporter family are mapped to the corresponding position in the DAT, identified by colored spheres of their respective Ca atom. Most residues cluster in a ring structure close to are at the membrane exposed surface of the transporter.

We propose a model that accounts for both, dominant and recessive transmission and also makes testable predictions about the actions of pharmacochaperones and chemical chaperones (Fig. 1). The model posits that SLC6 transporters engage luminal proteinaceous chaperones (e.g., calnexin, CNX in Fig. 1). In addition, the C-terminus engages a cytosolic heat-shock-protein relay (HSP in Fig. 1). Upon release of lumenal chaperones, the transporters oligomerize, adopt their final stable conformation and release the heat-shock protein from the C-terminus. This renders the binding site for SEC24C accessible and allows for ER export of the folded transporter. Conversely, if the transporter fails to reach a stably folded conformation, it is eventually subjected to ubiquitination (via an unidentified E3-ligase), dislocated/retrotranslocated and subjected to ER-associated degradation (left hand part in Fig. 1). The model is adapted from that put forth to explain trafficking of the A_2A_-receptor through the early secretory pathway [47] and is based on the following – albeit circumstantial – evidence:

(i) Oligomer formation is required for ER export of SLC6 transporters. Oligomerization-deficient mutants are retained in the ER [48,49]. Mutants, in which the C-terminus is truncated and thus the SEC24-binding site eliminated, act as dominant negative and retain the wild type transporter in the ER [50]; the same is true for fragments of the transporter [51].
(ii) In the ER, GAT1, SERT and DAT are engaged not only by the lumenal (i.e. Ca^2+^-binding lectin) domain of lectin, the transmembrane domain also shields the protein [52]. This peptide-based interaction is most pronounced with an oligomerization-deficient version of GAT1 [52]. Conversely, coexpression of lumenal chaperones increases the production of mature SERT in heterologous expression systems up to 3-fold [53].
(iii) The binding site for the SEC24 (the cargo receptor of the coatomer-II/COPII complex) resides in the C-terminus of GAT-1 [54], SERT and other transporters such as NET, DAT, GLYT1 and GAT3 [55,56].
(iv) In SERT, discrete mutations in the C-terminus, including the SEC24 binding site, result in folding defects [57]. Presumably, this phenotype reflects an acquired trait: bacterial transporters are inserted directly into their target membrane (i.e., the inner membrane of bacteria). Hence, they do not require any C-terminal quality control. In contrast, eukaryotic transporters are synthesized in the ER. Prior to reaching their target membrane (i.e., the cell surface), they must undergo quality control to preclude the delivery of incompletely folded proteins to the plasma membrane. Accordingly, it is not surprising that the C-terminus plays a role in folding. In fact, the recently solved structure of an insect DAT shows that the C-terminus folds back and interacts with intracellular loop 1 [58]. We therefore posit that the C-terminus is first occupied by a heat-shock protein-relay (HSP40–HSP70). We therefore posit that the C-terminus is first occupied SEC24. The chemical chaperone 4-phenylbuytrate (4-PB), which alters the expression of heat-shock proteins in a differential way and thus affects the heat-shock protein relay, increases the expression of SERT [59].
(v) Predictably non-specific chemical chaperones such as 4-PB [59], dimethylsulfoxide (DMSO) and glycerol effectively rescue mutants of SERT [58]. In contrast, pharmacochaperoning shows exquisite specificity: the only effective ligand which works is ibogaine (CID 442108) [59]. This indicates that the folding trajectory proceeds via the inward facing conformation, because ibogaine binds selectively to and stabilizes the inward facing state in the transport cycle [60]. In fact, the inward facing state is actually also favored by the ionic gradients in the ER.

Based on this hypothetical model, the following testable predictions can be made:

(i) Recessive mutations are stalled in a monomeric state. Their expression may be increased by interfering with ERAD, with lumenal and cytosolic chaperones (i.e., inhibitors of HSP70 and/or HSP90) and possibly by the addition of pharmacochaperones.
(ii) Autosomal dominant mutations are stalled in a dimeric state: depletion of calcium (e.g., by thapsigargin to suppress functional calnexin) ought to be irrelevant to their ER export, but compounds that bind to and stabilize the inward facing conformation ought to promote surface expression.
(iii) Finally, compounds that bind to the outward facing conformation are unlikely to act as pharmacochaperones. All inhibitors of SLC6 family members (but ibogaine) that are used either for therapeutic or recreational purposes bind preferentially to the outward facing conformation. It is therefore not surprising that upregulation of transporters has not been observed upon chronic administration of tricyclic antidepressants, SSRIs, cocaine, etc.

### 1.3 A microscopic model for folding of SLC6 family members

**Fig. 3:**
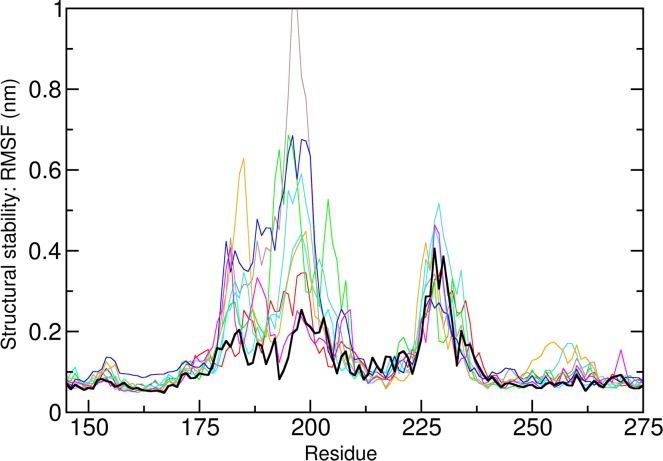
Structural stability analysis the extracellular loop 2. Fluctuations (quantified as Root Mean Square Fluctuations or RMSF) around the equilibrium position contain information on structural stability. The larger the RMSF, the larger the fluctuations and therefore the lower the structural stability. The black line shows the RMSF of the properly folded DAT transporter, calculated for a 20 ns long trajectory. Ten additional trajectories of model, where the first part of the extracellular loop 2 between residue 173 and residue 202 was not properly modeled show consistent higher RMSF values. The regions of low RMSF coincide with helical structure elements.

The acid test for our understanding the folding trajectory is that it can be simulated. Structures are available for LeuT*Aa* in several conformations. In addition, the structure of an insect dopamine transporter has recently been solved in an inhibitor-blocked outward-facing conformation [58]. The folding problem may therefore become amenable to computational approaches. It is obviously impossible to reach the folded structure from the primary sequence but it should be possible to determine which forces govern the assembly of helical transmembrane segments, how charges are best shielded from the lipid environment when helices are released from the SEC61 channel into the lipid environment of the membrane and how the final stable conformation is stabilized by those segments that are exposed to the aqueous milieu. This includes the intracellular and extracellular loops and the C-terminus; the N-terminus appears dispensable, because it can be truncated without impairing surface expression [61]. As a starting point, the problem may be approached by examining the effect of mutations that are known to preclude surface expression by causing ER retention and subsequent degradation of the protein. Alternatively, it is also possible to examine the effect of thermostabilizing mutations [62]. The effect of mutation or imperfect structural models can be directly studied using molecular dynamics simulations. An example of such an exercise is shown in Fig. 3: using a model of DAT, we studied the stability of the transporter in a membrane environment [45]. The template LeuTAa used for model building has a much shorter extracellular loop 2 (EL2) and cannot serve as a template for this loop. In the new drosophila DAT used for crystallization a large part of this loop was removed. Fig. 3 shows a structural mobility analysis (fluctuation around the equilibrium conformation quantified as Root Mean Square Fluctuation or RMSF) of the EL2 (residue 172–237) from 20 ns long simulations. The correctly folded loop (shown in black) has a substantially lower mobility in the region of the EL2, where the LeuTAa template could not serve as a template (residue 173–202). The first part involves a highly conserved tryptophan residue (W184 in DAT). If mutated, surface expression is abolished [63]. Trajectories showed large mobility and an unstable loop, when this tryptophan was not properly positioned. Residue H193 of DAT is part of a zinc binding site. Only if properly assembled, the residues coordinating remain next to the zinc ion. Otherwise, they undergo large movements.

### 1.4 ABC-transporters

ATP binding cassette (ABC) proteins are found in all extant phyla. In humans 48 genes code for ABC proteins, which on basis of sequence similarity have been classified in seven subfamilies. Most of these ABC proteins have been shown to be involved in the movement of cargo across biological membranes, but other functions include ion conductance and ion channel regulation. Several ABC transporters, primarily P-glycoprotein, multidrug resistance protein and breast cancer related protein are involved in drug transport and therefore play a key role in drug disposition. 22 ABC-proteins have been linked to more than 27 human disease etiologies, whereby the malfunction of a single ABC protein occasionally leads to more than one disease entity [64].

### 1.5 Several ABC exporters share a common fold

**Fig. 4:**
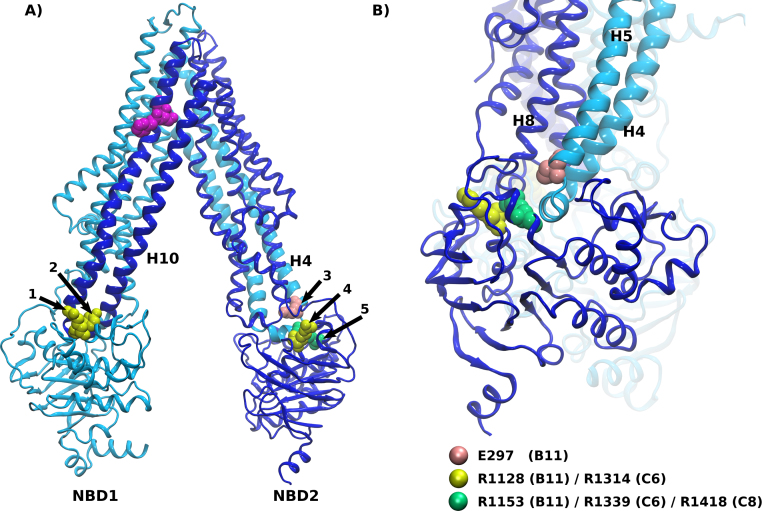
Mapping of selected folding deficient mutations of the ABC transporter family on a structural model of the inward facing conformation of human P-pg (template *C. elegans* ABCB1 (PDB ID: 4F4C)). The N-terminal half of the transporter is colored in cyan, the C-terminal half in dark blue. Panel A shows the paradigmatic fold first reported for SAV1866 and subsequently found to be shared with MsbA, mouse mdr1a, and human ABCB10. Fully assembled transporters show a domain swapped architecture whereby intracellular loop 2 (connecting helices 4 and 5 of TMD1) interacts with NBD2, while ICL4 of TMD2 interacts with NBD1. Residues 951–953 photolabeled by propafenones binding to the transporter in mode 2 are highlighted in magenta. Location of selected disease causing mutations are shown in pink yellow and green in VDW rendering. The residue shown in pink, yellow and green are found mutated in one, two or three ABC transporter, respectively. All disease causing mutations are projected into the P-gp homology model based on sequence alignments. 1: deltaF490 (ABCB1), dF508 (ABCC7), 2: I507 (ABCC7), Q141K (ABCG2); 3: E297G (ABCB11); 4: R1128 (ABCB11) and R1314 (ABCC6) and 5: R1153 (ABCB11), R1339 (ABCC6) and R1418 (ABCC7). Panel B shows a close up of the transmission interface in a side view of NBD2. Positions of mutations 3, 4 and 5 can be appreciated to affect either intracellular loop 2 or NBD2. (For interpretation of the references to color in this figure legend, the reader is referred to the web version of the article.)

In recent years higher resolution crystal structures of ABC exporters have become available. These include SAV1866, a multidrug transporter from *S. aureus* [65], *C. elegans* ABCB1 [66], mouse mdr1a [67], the human ABCB10 transporter [68] and the lipid A transporter MsbA [69]. These structures indicate a common architecture of several prokaryotic and mammalian transporters, including the ABCB subfamily members. This paradigmatic fold, which was first described for SAV1866 [65] and subsequently confirmed for other ABC exporters shows a remarkable hallmark. In contrast to the side-by-side arrangement of TMDs and NBDs observed for ABC importers, efflux transporters show a domain swapped architecture. While the first intracellular loop of each TMD forms contacts with the ipsilateral NBD, intracytoplasmic loop 2 of each half reaches over to engage in interactions with the contralateral NBD. Contacts are formed via four short a-helices of 2–3 helical turns, the axis of which runs in parallel to the membrane-oriented surface of the NBDs. These helices, termed “coupling helices” by Dawson and Locher [65] and Hollenstein et al. [70] contribute to formation of the transmission interface, which connects the nucleotide binding domains and the transmembrane domains. Coupling helices of the second intracellular loops come to rest in a groove between a-helical and core domain of the NBD on top of a ß-strand, which precedes a loop region containing the eponymous glutamine of the so called Q-loop, a hypothetical sensor of cleavage of the ATP ß-? anhydride bond (Fig. 4). The rigid body movement between a-helical and F1 type core domain of the respective NBD can be envisioned to affect positioning of these second coupling helices, which in turn act as levers transmitting the conformational change induced by ATP binding and hydrolysis to the membrane spanning portion of the transporter. Correct positioning of ICLs 2 and 4 as outlined above, can be assumed to represent a central step in adopting a fully folded state and any mutations of residues that lie close to this interface might turn it into an Achilles heel that affects architecture of ABC-transporters on a larger scale. Although folding trajectories are only partially understood, cornerstones of the folding process have been defined: (i) As illustrated above in the discussion of the Levinthal paradoxon, sampling of all possible conformational states is not a viable biological option for achieving the correctly folded state of a protein. Rather, a sequence of timed events along folding trajectories is required to occur during the production of a correctly folded and functional protein. (ii) Folding of individual domains precedes positioning of these domains relative to each other (also referred to as superfolding). Evidence suggests that translational pausing at rare codons might provide a time delay to enable independent and sequential folding of defined portions of the nascent polypeptide chain, i.e. rare codons are frequently found at C-terminal ends of domains or subdomains. These provide a short respite period for folding, before synthesis of the protein chain of subsequent domains is initiated [71]. For a half transporter consisting of two monomers, each containing a single TMD and NBD, insertion of coupling helix 2 in the NBD of a second monomer is likely to require completion of the synthesis of the entire monomer. Similarly, for full transporters, it might be assumed that completion of the entire sequence is likely to precede engagement of ICLs2/4 in the respective NBDs of the other half of the transporter. (iii) Conformational changes at the transmission interface of an ABC transporter are a prerequisite for function. On the other hand, folding requires defined interactions at this interface, which are able to confer necessary stability. The following lines of evidence indicate that interactions at the transmission interface are critical: (i) Different ABC transporters show missense mutations in similar or identical positions along this domain interface, which are capable of inducing incomplete folding. These mutations frequently affect the NBD a-helical and core domains as well as the intracellular contact loops in regions that are either close to the coupling helices or lie within them. Table 2 contains a list of representative mutations, their location within the protein and diseases associated with these mutations.

A similar clustering of disease-linked mutations has been observed for G-protein coupled receptors [84], suggesting that mutations in protein families occur in structurally similar or even identical positions. These positions are prone to result in malfunction, which is rooted in the common fold (molecular architecture) of the protein family. Only secondary to this underlying fold-determined cause of disease is the associated phenotype, which is determined by the physiological role that the individual member of the protein family plays. The word “phenotologous sites” or “disease phenotology” has been suggested by the group of Sanders to signify this circumstance [84]. (ii) Active site compounds rescue folding deficient mutants in the a-helical domain of the NBDs. The distance between the active site and the mutated residue in the NBD of approximately 60 Å indicates a long-range effect (Fig. 4). Mechanistically, the ability of active site compounds to rescue NBD mutants can be explained by the fact that both the formation of the active site and the transmission interface are contingent on correct positioning of the second intracytoplasmic loops (Spork, Sohail, Chiba, in preparation). (iii) Mutations in the active site lead to decreased binding affinity of active site compounds and decreased ability to rescue trafficking (Spork, Sohail, Chiba, unpublished).

### 1.6 Candidates for trafficking rescue in the family of ABC proteins

It has been well appreciated that only a fraction of wild type CFTR (cystic fibrosis transmembrane conductance regulator) reaches the membrane of airway epithelial cells and exocrine glands and that CFTR type 2 mutations lead to an even further impairment of trafficking [85–88]. In recent years evidence has accumulated that not only folding of CFTR, but also of other ABC proteins is frequently affected by nonsynonymous mutations and that incomplete folding is related to a number of human diseases. These include ABCA1 (HDL-deficiency, Tangier disease) [89,90], ABCB4 (progressive familial intrahepatic cholestasis type 3; PFIC3) [91], ABCB11 (PFIC2) [92], ABCC2 (Dubin-Johnson syndrome) [93,94], ABCC6 (pseudoxanthoma elasticum, general arterial calcification of infancy) [95] and ABCG2 (gout) [96]. A systematic survey of non-synonymous mutations indicates that misfolding as an underlying mechanism of ABC protein deficiencies might be more prevalent than expected. Evidence for the effect of mutations and non-synonymous SNPs on protein trafficking, maturation, or ER associated degradation has been provided for at least the following members of the human ABC family: ABCA1, ABCA3, ABCA4, ABCB1, ABCB4, ABCB11, ABCC2, ABCC4, ABCC7, ABCC8, ABCC11 and ABCG2 (reviewed in Nakagawa et al. [97]). Undoubtedly, ubiquitin-mediated proteasomal degradation of ABC transporters due to incomplete folding is recognized as an important pathogenetic principle for diseases associated with ABC protein deficiency beyond the well-appreciated paradigm of cystic fibrosis.

### 1.7 Rescue of trafficking by chemical and pharmacological chaperones

Because of the recent successful market introduction of a small molecule potentiator of CFTR (Ivacaftor, VX-770) [98], combinations with a pharmacological chaperone (Lumacaftor, VX-809, CID 16678941) are now studied in a clinical setting in patients carrying the frequent deltaF508 allele [99]. Because of a large number of ongoing industrial and academic ventures focusing on the development of folding correctors for CFTR, a separate article in this issue is dedicated particularly to the treatment of cystic fibrosis.

Low molecular weight compounds with an ability to rescue trafficking deficient ABC proteins other than CFTR have been identified. These include chemical chaperones such as DMSO, glycerol, trimethylamine-*N*-oxide (TMAO) and 4-phenyl butyrate (4-PB). The latter is approved as a therapeutic agent for disorders of the urea cycle and used as a candidate chemical chaperone in clinical studies. A recent case study indicates the therapeutic potential of 4-PB to improve the clinical condition of a patient suffering from progressive familial intrahepatic cholestasis type II, caused by a folding deficiency of the bile salt export pump [100].

Specific and nontoxic pharmacological chaperones can be used at much lower concentration than chemical chaperones. In vitro experiments suggest that breast cancer related protein (BCRP) [101], CFTR (ABCC7) [102,103], MRP1 (ABCC1) [104], ABCB1 and SUR1 (ABCC8) can be rescued by small molecules.

Misfolding/incomplete folding is thus a significant cause of disease and development of small molecules that correct surface expression and by that enhance transport activity, has been initiated for several ABC proteins. These include the bile salt export pump (BSEP, ABCB11), which is rate limiting for bile flux. Both, natural bile acids, in particular chenodeoxycholic acid (CDCA, CID 10133) and the non-steroidal farnesoid-X-receptor (FXR) agonist GW4064 (CID 9893571), as well as derivatives lacking FXR-agonist activity were able to enhance function of the trafficking deficient BESP mutant E297G, likely by increasing the amount of protein at the plasma membrane [105–108].

Similarly, rescue of the folding deficient ABCB4 variant I541F, a phospholipid transporter associated with progressive familial intrahepatic cholestasis type 3, by cyclosporine A (CID 5284373) has been reported in an in vitro setting [75]. An additional transporter, which may be amenable to pharmacochaperone treatment is ABCC6. Mutations in this transporter are causally related to aberrant trafficking, giving rise to a disease known as pseudoxanthoma elasticum [95]. Furthermore, pharmacochaperones have been suggested as a treatment option in gout cases caused by incomplete folding and decreased functional activity of the ABCG2 variant Q141K [96]. Here, compound VRT-325 (CID 11957831), initially discovered in a high-throughput screen to identify molecules that would correct mutant CFTR, was shown to increase levels of mature fully glycosylated protein.

Notably, some of these studies indicate that small molecules, which bind to the membrane spanning portions of the transporters, are capable of correcting mutations in spatially separated intracellular domains and NBDs. The membrane spanning portion confers substrate specificity and thus represents the most diverse regions in multiple sequence alignments of the protein family. Hence, expectations are tied to the use of substrate analogs or derivatives that address these diverse substrate sites of the transporters and exploit their capability of exerting long range corrector effects within the protein to keep off target effects to a minimum. It is, however, inevitable that some of these correctors will also elicit untoward effects by acting as substrates and/or inhibitors of the very transporter that they are designed to rescue. In addition, blockage of related transporters is to be anticipated, because several ABC transporters have overlapping substrate specificities. However, these problems may be obviated by using correctors that have short half-lives. Other, strategies are also conceivable (i.e., allosteric correctors).

## 2 Concluding remarks

Rescue of trafficking deficient mutants by either chemical or pharmacological chaperones has emerged as a promising treatment concept for monogenic diseases associated with SLC6 and ABC transporter malfunction. ER-checkpoints equate correct folding to proper function. Using this surrogate, they efficiently screen about 6000 membrane proteins (encoded by the human genome) and assure their quality prior to their further processing along the secretory pathway. The concept that folding of mutated proteins may be corrected by small molecules relies on the assumption that the reverse equation (i.e., impaired folding = inactive protein) does not always hold true. While mutant proteins are in their nascent state, pharmacochaperones may escort them past checkpoints of the ER quality control system and by that allow them to reach their final cellular destination. Indeed experimental evidence has been provided that residual function is associated with a larger number of folding deficient transporter mutants and that this residual function may suffice to alleviate disease symptoms. Though mutations affecting functional motifs of transporters cannot be corrected, available evidence indicates these mutations to be by far outnumbered by non-synonymous mutations in similar positions of structurally related members of protein families. We therefore propose that critical regions in the 3D structure of SLC6 and ABC transporters exist, which represent Achilles’ heels in the folding process. If, as indicated, a common structural basis indeed exists, correctors should be expected to show activity in more than one folding deficient mutant.

## Figures and Tables

**Table 1:** Mutations in human SLC6 transporters.

Gene	Protein name	Candidate pathogenic coding variants/missense mutations^a^	Disease	Change of function	Altered expression
SLC6A1	GAT-1	?	*?*	?	?
	(GABA transporter-1)				
SLC6A2	NET (norepinephrine transporter)	F528C	Orthostatic intolerance/postural hypotension	Reduced affinity for desipramine [8,9]	
		R121Q, N292T, A369P, A457P, I549T	Orthostatic intolerance/postural hypotension		Reduced surface expression/ ER retention; (autosomal dominant) (A457P) [8,9]
SLC6A3	DAT (dopamine transporter)	V382A	ADHD (attention-deficit hyperactivity disease)		Reduced surface expression [10]
		A559V, T356M,		Transport cycle mutant (exaggerated efflux) [11–13]	
		R615C		altered microdomain/ flotillin association [14]	
		V158F, L224P, G327R, L368Q, P395L, R521W, P529L, P554L	Childhood (recessive) parkinsonism-dystonia		ER-retention, reduced surface expression [15,16]
SLC6A4	SERT (serotonin transporter)	G56A	Autism, Asperger syndrome and obsessive-compulsive disorder^b^		Enhanced expression [17]
		I425V, I425L, F465L, L550V, K605N			I425V: enhanced uptake/gain of function [17–19]; other mutations not characterized in functional assays
SLC6A5	GLYT2 (glycine transporter-2)	>15 mutations: e.g. A89E, A275T, L306V, W482R, S510R Y705C	Hyperekplexia/startle disease [21–23]		ER-retention/reduced surface expression [21,23,24]
SLC6A6	TAUT (taurine transporter)	?	? Suspected involvement in retinal degeneration based on murine model		
SLC6A7	PROT (brain-specific proline transporter)	?	? Gene deletion suspected to contribute to intellectual disability [25]		
SLC6A8	CT1 (creatine transporter-1)	>21 missense mutations	X-linked mental retardation [26–29]	No mutation documented to lead to ER retention	
SLC6A9	GLYT1 (glycine transporter-1)	?	? Suspected involvement in glycine encephalopathy/non-ketotic hyperglycinemia based of murine model		
SLC6A11	GAT3 (GABA-transporter-3)	?	? Polymorphism associated with neuroleptic-induced tardive dyskinesia [30]		
SLC6A12	BGT1 (betaine-GABA transporter-1)	?	? Polymorphisms association with negative symptoms in schizophrenia [31] and aspirin-intolerant asthma [32]		
SLC6A13	GAT2 (GABA-transporter-2)	?	? Polymorphisms association with chronic kidney disease [33]		
SLC6A14	ATB0 transporter for neutral, cationic amino acids	?	? Polymorphisms association with increased risk for meconium ileus in newborns with cystic fibrosis [34] and with obesity [35,36]		
SLC6A15	B(0)AT2 transporter for large neutral amino acids	T49A, K227N, L260P^c^, G268R^c^, D278V^c^, A400V, L421P, I500T, N591D, A601T, E684D, G710R	Association with increased risk for major depression	T49A and A400V result in increased proline uptake) [37]	None of the variants impede surface expression
SLC6A16	NTT5 orphan sodium- and chloride-dependent neurotransmitter transporter	?	?	?	?
SLC6A17	NTT4/XT1 synaptic vesicle neutral amino acid transporter	?	?	?	?
SLC6A18	B0AT3 renal neutral amino acid transporter/glycine transporter	G79S, L478P [should probably be P478L mutation], G496R	Contribution to iminoglycinuria/hyperglycinuria if one allele of SLC36A2 is inactivated [38]		No evidence for trafficking defect of L478P but greatly reduced surface expression of G79S and G496R
SLC6A19	B0AT1 renal and intestinal amino acid transporter	R57C, A69T, D173N, L242P, P265L, G284R, S303L, E501K	Hartnup disease (dermatitis and seizures) [39–41]		D173N, L242P, E501K-reduced activity/surface expression; surface expression contingent on interaction with collectrin/ACE-2 (angiotensin-converting enzyme-2) [42,43]
SLC6A20	XTRP3 proline imino transporter	T199M	Contributes to iminoglycinuria if one allele of SLC36A2 is inactivated [38]	Impairs transport but not surface targeting [38]	

a Mutations that result in frame shifts and/or premature termination, changes in splice acceptor sites, etc. are not listed.

b The monogenic association of the I425V mutation with obsessive-compulsive disorder has been questioned [20].

c Residues of isoform 2 that has a different sequence between residue 253 and 289; downstream residues are missing, effectively truncating the sequence after transmembrane helix 5.

**Table 2:** Mutations in ABC transporters.

Gene	Protein name	Pathogenic coding variants	Phenotype
ABCA1	CERP (cholesterol efflux regulatory protein)	A1046D (C-term. a-helix of NBD1) [72] R2004K (N-term. a-helix of NBD2) [73]	Tangier disease (HDL deficiency)
ABCA3		N568D (F1-type core domain, NBD1) [74]	Neonatal respiratory distress syndrome
ABCB4	MDR3	I541F (C-term. a-helix of a-helical domain, NBD1) [75]	PFIC3 (progressive familial intrahepatic cholestasis type 3)
ABCB11	BSEP (bile salt export pump)	E297G (coupling helix 4, TMD2) [76] D482G (antiparallel domain of NBD1) [76]^a^	PFIC2 (progressive familial intrahepatic cholestasis type 2)
ABCC2	cMOAT (canalicular multispecific organic anion transporter)	R768W (N-term. a-helix of aa-helical domain of NBD1) [77] A1450T (N-term. a-helix of a-helical domain of NBD2) [78]	Dubin–Johnson syndrome
ABCC6	MOAT-E (multispecific organic anion transporter)	R1138Q (coupling helix 4, TMD2) [79] R1339C (F1-type core domain NBD2) [79]	Pseudoxanthoma elasticum
ABCC7	CFTR (cystic fibrosis transmembrane conductance regulator	?F508 [reviewed in 80]^b^ ?I507 (N-term. a-helix of a-helical domain of NBD1) [reviewed in 80] N1303 (N-term. a-helix of a-helical domain of NBD2) [reviewed in 80] L1065P (coupling helix 4, TMD2) [reviewed in 81]	Cystic fibrosis
ABCC8	SUR (sulfonyl urea receptor)	R1493W (C-term. a-helix of a-helical domain NBD2) [82]	Familial hyperinsulinism
ABCG2	BCRP (breast cancer resistance protein)	Q141K^c^ (N-term. a-helix of a-helical domain of NBD1) [83]	Gout

This table lists representative examples of phenotypes, which are characterized by decreased surface expression. The listing is anecdotal, but in a representative way illustrates the importance of a correctly formed transmission (TMD/NBD) interface for correct folding and trafficking. A comprehensive publication comprising more than 120 folding deficient mutations is in preparation.

a Most frequent mutants in European population with close to 60% of patients harboring either one or both variants.

b Most frequent mutation found in at least one allele of 90% of cystic fibrosis patients.

c Allelic frequency of about 30% in the Asian population.
